# Identifying individuals using proteomics: are we there yet?

**DOI:** 10.3389/fmolb.2022.1062031

**Published:** 2022-11-29

**Authors:** Ivo Fierro-Monti, James C. Wright, Jyoti S. Choudhary, Juan Antonio Vizcaíno

**Affiliations:** ^1^ European Molecular Biology Laboratory, European Bioinformatics Institute (EMBL-EBI), Wellcome Trust Genome Campus, Hinxton, United Kingdom; ^2^ Institute of Cancer Research, London, United Kingdom

**Keywords:** proteogenomics, amino acid variants, protein variants, omics data analysis, RNA editing, proteomics data, genomics data, identifiability

## Abstract

Multi-omics approaches including proteomics analyses are becoming an integral component of precision medicine. As clinical proteomics studies gain momentum and their sensitivity increases, research on identifying individuals based on their proteomics data is here examined for risks and ethics-related issues. A great deal of work has already been done on this topic for DNA/RNA sequencing data, but it has yet to be widely studied in other omics fields. The current state-of-the-art for the identification of individuals based solely on proteomics data is explained. Protein sequence variation analysis approaches are covered in more detail, including the available analysis workflows and their limitations. We also outline some previous forensic and omics proteomics studies that are relevant for the identification of individuals. Following that, we discuss the risks of patient reidentification using other proteomics data types such as protein expression abundance and post-translational modification (PTM) profiles. In light of the potential identification of individuals through proteomics data, possible legal and ethical implications are becoming increasingly important in the field.

## Introduction

Human phenotypes play a key role in biomedical research and clinical practice towards better diagnosis, patient stratification and the selection of effective treatment strategies. Computational approaches developed for the integration of multiple omics data types allow for a more holistic understanding of molecular mechanisms in health and disease-related processes (Chen et al., 2012). Such combined approaches can lead to the discovery of biomarkers that enable personalised medicine approaches by representing personalised prognosis and treatment efficacy. The acquisition of data derived from genomic, transcriptomic, and proteomic personal phenotypes (among other types of omics techniques) holds implications for both personalised medicine and data privacy-related issues, considering technical, ethical, and legal aspects.

Research data collected as part of biological research can raise data privacy issues when: 1) the data contains protected patient information or otherwise when the data can be linked to a single individual; 2) data need to comply with laws and/or regulations regarding data privacy (e.g., the General Data Protection Regulation in the European Union); and/or 3) the informed consent forms from patients include limitations on data sharing (Bandeira et al., 2021). The question of whether it is possible or not to identify individuals using omics data has already been thoroughly explored for DNA/RNA sequencing information (Gymrek et al., 2013; Erlich & Narayanan, 2014), which is thus generally considered to be patient-identifiable information by data protection regulations. This is an extensively reviewed topic including e.g., literature devoted to the state-of-the-art in genomic data privacy (Malin, 2005), breaching and protection of privacy (Erlich & Narayanan, 2014) (Naveed et al., 2015), technical approaches to address privacy in genomics (Wang et al., 2017), and privacy-enhancing technologies in genomics [PoPETs Proceedings (petsymposium.org)], among other topics.

With the growing importance of clinical proteomics studies, potential ethical and legal issues are becoming increasingly relevant in the field (BOONEN et al., 2019). Data generated from proteomics studies include several data types that can be characteristic of the proteomes of individuals at a given time. These can include e.g., amino acid sequences, protein expression levels and PTM (post-translational modification) profiles.

The need for tailored data management practices for sensitive human proteomics data (Bandeira et al., 2021), and the related ethical issues (Porsdam Mann et al., 2021) are topics that have been recently reviewed. In this minireview, we describe the current state-of-the-art when it comes to identifying individuals based solely on proteomics data. Protein sequence variation information will be covered in-depth, along with other proteomic data types.

### Types of genetic variation and its consequences at the protein and the phenotype level

**FIGURE 1:**
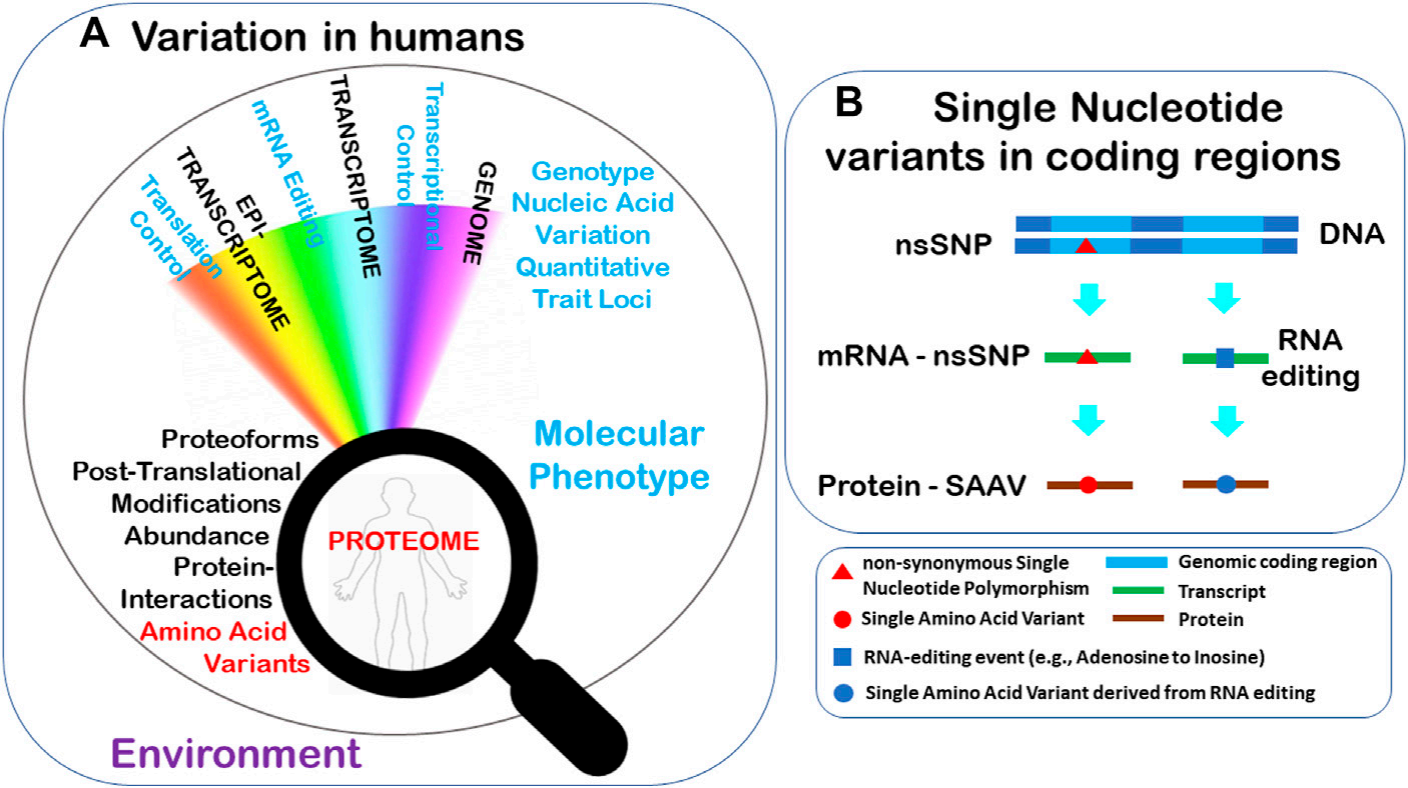
**(A)** Many downstream processes may be impacted by genetic variation in addition to the Environment and may implicate an individual’s proteome and phenotype. **(B)** Two examples of sequence variants represented by common genome nsSNPs and epitranscriptomic RNA editing lead to changes in the protein sequences.

Genomic sequence variation and epigenetic modifications may affect downstream processes in numerous ways, including changes to the proteomes and phenotypes of individuals (Figure 1A). There are various types of genomic variation events being the most common ones: single nucleotide polymorphisms (SNPs) (Figure 1B), insertions and deletions (INDELs) and larger genomic structural rearrangements. Sequence variation residing within, or outside gene coding (exons) can potentially manifest at the protein level in a variety of ways. Synonymous SNPs (those not altering the protein amino acid sequence), can potentially affect the regulation of gene expression at the transcriptional or translational level. Genomic variants may also be non-synonymous (nsSNP), directly modifying the amino acid sequence encoded by the gene, or in the case of RNA editing (Rengaraj et al., 2021), the transcript (Figure 1B). There are different types of nsSNPs, ranging from a missense variant causing a Single Amino Acid Variant (SAAV), to much larger changes such as multiple amino acid insertions, deletions, protein truncation (nonsense variants), coding frameshifts, protein mis-splicing and read-through, and gene fusion events (Vegvari, 2016). Transcript variants, substitutions arising from RNA-editing that recode protein sequences which consequently may alter the PTM profile of the recoded residues increase proteome diversity. Genomic variants result in the synthesis of different proteoforms, which can also affect PTMs, contributing to the proteome’s complexity and variability (Smith et al., 2013), as well as potentially altering organisms’ phenotypic characteristics. It should also be noted that each gene is present in two copies or alleles in diploid organisms such as humans, and variants may arise in either one (heterozygous) or both (homozygous) alleles. When an nsSNP is heterozygous, allele-specific protein expression and bias can occur.

### Strategies to detect sequence variation at the protein level

Thanks to the application of mass spectrometry (MS) high-throughput proteomics approaches (Aebersold & Mann, 2016), it is now possible to systematically analyse “proteotypes,” which can be defined as a state of a proteome associated with a specific phenotype. The most common shotgun proteomic approach is liquid-chromatography (LC) followed by MS or also known as bottom-up proteomics, where the peptides resulting from digested proteins are separated by LC before being analysed by the MS instrument. The so-called top-down proteomics techniques directly analyse intact proteins, allowing precise and comprehensive characterisation of various proteoforms, such as protein mutations from genetic polymorphisms and RNA splice variants as well as PTMs. This approach potentially delivers data including a comprehensive characterisation of proteoforms (Smith et al., 2013; Smith and Kelleher, 2018). However, currently, there are many technical challenges for top-down proteomics technologies, such as obstacles in the characterisation of high-molecular-weight and low-abundance proteins, and the lack of bioinformatics tools for the analysis of complex proteoform MS data (Schaffer et al., 2019). These technical limitations in depth and throughput hamper its wider application (Lin et al., 2021). Bottom-up proteomics adds an additional layer of complexity, due to the required inference of peptides (including peptide variants) to the parent proteins. An alternative to shotgun proteomics which tests a random portion of the proteome, Selected Reaction Monitoring (SRM) target assays, use optimised and specific separation and detection parameters for a subset of preselected or proteotypic peptides. Targeted MS considerably improves consistent and precise quantification over standard shotgun LC-MS and provides the sensitivity and selectivity required to detect and precisely quantify low-abundance proteins and proteoforms such as protein variants [reviewed in Schmidt & Schreiner, 2022]. Moreover, one of the main constraints of targeted proteomics is limited multiplexing. This has recently been addressed effectively by allowing the simultaneous quantification of about 1,000 analytes in one analysis (Stopfer et al., 2021). Additionally, two types of data acquisition exist. In Data Dependent Acquisition (DDA) approaches, only the most abundant peptide ions are selected for fragmentation. To overcome this issue (which potentially leads to a smaller proteome coverage and consequently, to fewer SAAVs identified and quantified) a systematic selection of the whole range of ion peptide masses can be performed in cycles before the fragmentation (in Data Independent Acquisition approaches [DIA]). Here, the analysis usually relies on spectral libraries. These libraries can be generated experimentally but also using artificial intelligence algorithms. Their use in DIA analysis is an active topic of research (Searle et al., 2020; Sun et al., 2022).

Proteogenomics (PG) approaches can be used to integrate DNA/RNA sequencing (genomics, RNA-Seq, Ribo-seq) and proteomics data so that genetic variation in the protein amino acid backbone can be detected. DNA/RNA sequencing data from the same samples are used in PG approaches to identify expressed protein variants (Nesvizhskii, 2014) for known genomic variation events. Less prevalent and more technically challenging methods for the detection of protein sequence variation include *de novo* peptide sequencing, spectral library searching and open modification searches (OMS). First, *de novo* peptide sequencing is a method in which a peptide sequence or a partial sequence tag can be determined directly from the MS/MS spectra. This overcomes the limitation of traditional database searching methods to only being able to assign peptides to spectra if the sequence is present in the database. However, this strategy is more computationally demanding and heavily reliant on spectra quality and good fragmentation of peptides. Often *de novo* approaches are used in conjunction with sequence database searching. Another approach without using a reference sequence database is spectral library searching, where experimental spectra are matched to a spectral library (Shao & Lam, 2017). A traditional limitation of this approach is that spectral libraries require the previous assignment of the mass spectra to peptide identifications. Finally, Open Modification Searches (OMS), enable searching for PTMs and SAAVs by considering potentially all mass shifts of an unmodified peptide. OMS can identify many spectra left unassigned in a standard database search. However, the vast increase in search space causes a significant loss in sensitivity and an increased false discovery rate (FDR).

**FIGURE 2:**
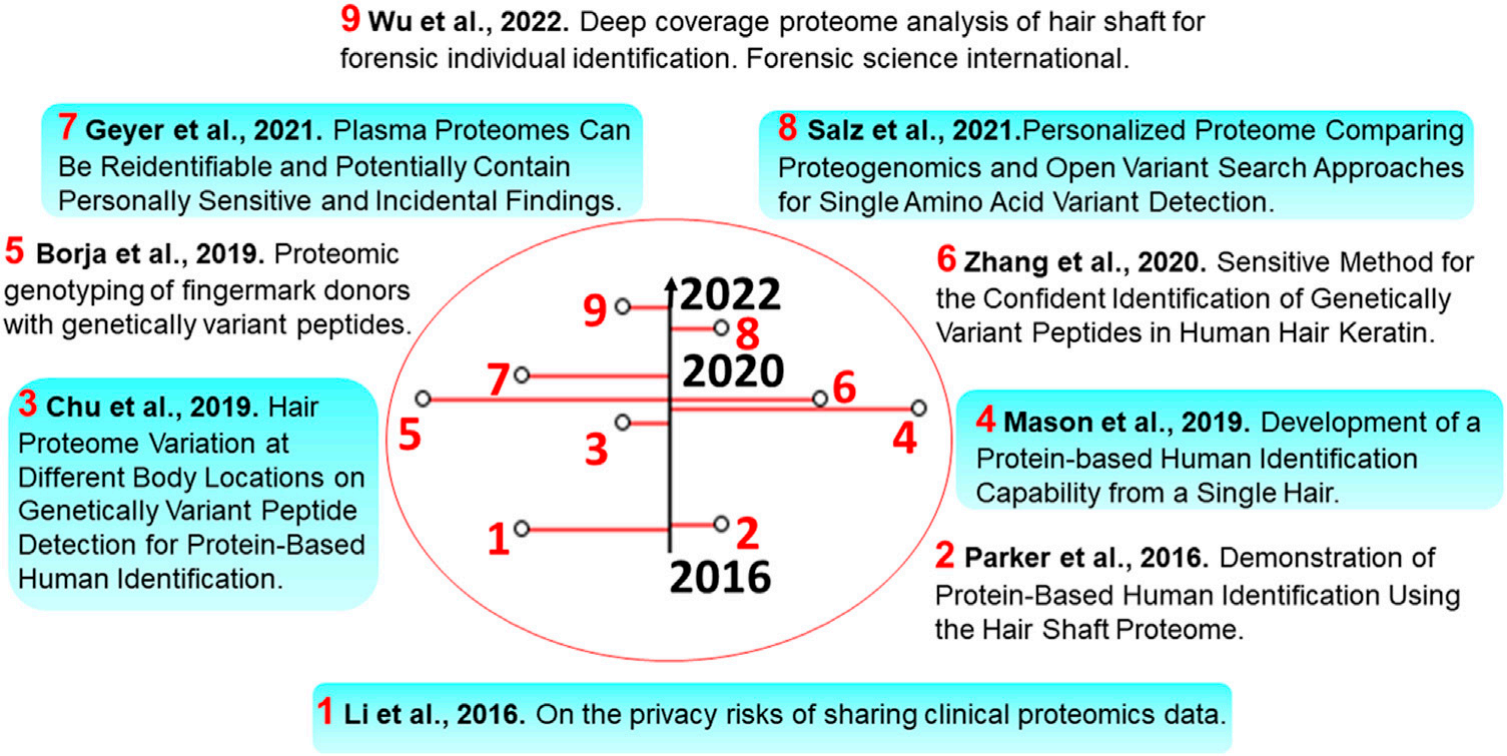
Timeline of selected publications in this manuscript. Some timely advancements toward identifying SAAVs and individuals’ proteomes are described in the illustration.

A recent comparison between the PG and OMS approaches [Figure 2 text-box 8, Salz et al., 2021] showed that currently, PG represents a better alternative for the detection of variant peptides. In this study, seven times more variant peptides were detected by the PG method, whereas the OMS method produced many false-positive identifications not supported by the genome sequence. Additionally, large numbers of false negatives were also detected since it is often challenging to distinguish between the delta masses arising from PTMs or SAAV-containing peptides.

### Limitations in the detection of protein sequence variation using MS

Considering the current state-of-the-art in MS-based proteomics, a significant portion of the proteome remains undetected in any given proteomics study. This is due to different technical and biological reasons, which contribute to the limitations in the detection of peptide variants (a subset of the total proteome). These unobserved peptides will include existent peptide variants, thus reducing the risk of individual identification or reidentification.

1) Technical factors. In each sample, the dynamic range and peptide detectability in the mass spectrometer (due to the peptide ionisation properties) restrict the observable portion of the proteome. Moreover, some protein regions cannot be digested by trypsin (the most used protease), making them undetectable by the common proteomics DDA workflows. Non-specific adsorption loss of peptides during sample preparation may affect the detection of protein sequence variation. There are also limitations related to the instrumentation because not all peptides can be fragmented simultaneously. Additionally, the identification of peptide from the MS/MS spectra are typically performed using a protein sequence database analysis approach. In fact, *de novo* interpretation of MS/MS spectra (a database-free analysis method) is usually quite limited. Therefore, at present, for proteomics data to get an acceptable protein sequence coverage, a high-quality protein sequence database is generally required. However, due to the reasons explained above, it is not unusual that some proteins are identified by just one or two representative peptides, which results in a very low overall sequence coverage. This exemplifies a quite different scenario when compared with DNA sequencing technologies, which can usually provide very high sequence coverages (Bandeira et al., 2021).
2) Biological factors. It is also possible for genome sequence variation to go undetected in the proteome for a variety of biological reasons. First, many genomic variants can be found in intronic regions, which are not translated. Second, most amino acids are encoded by more than one codon hence a single nucleotide change in a protein-coding exon can be silent (synonymous). Third, a variant may also disrupt the translation process, resulting in a decrease in the abundance of the variant protein, which can make it undetectable. Fourth, it should be noted that only a portion of the entire proteome is expressed at any given time (Bandeira et al., 2021).

Considering all these technical and biological factors, in our view, the potential risk of identifying individuals from proteomic data alone is low, unless one considers the potential detection of rare sequence variants with a very low prevalence. To the best of our knowledge, there is no population-based SAAVs data derived from proteomics studies where SAAV frequencies have been estimated. However, in the case of human genotypes, the larger the sample size from which the allele frequencies can be obtained, the more independent genetic markers needed to identify individuals (Visscher & Hill, 2009), as may also be the case for the SAAVs that are necessary for individual identification. In a previous study, a panel of 50 SNPs was sufficient to identify an individual unambiguously: the probability of identity was 6.9 × 10^–20^ when assuming no family relations (Yousefi et al., 2018). In fact, if an allele is very rare, it can dramatically reduce the number of identifiable people with that given SAAV. It is also important to highlight that the risk of identification of individuals should be balanced with confidence in the peptide identifications, considering the different levels of FDR and other statistical scores. Indeed, since every peptide identification has different statistical scores derived from the identification process, some SAAVs are more statistically significant than others.

### Case studies related to the identifiability of individuals in proteomics data

Biomedical and forensic studies are two disciplines where the concept of personal proteomes is relevant in the context of identifiability. In both cases, the potential identifiability of individuals can lead to mainly incidental (coming from biomedical data) or intentional (forensic) findings. However, the number of studies in the literature addressing the identifiability risks in proteomics data is so far small.

First, in the context of applications in the forensic sciences, the detection of proteins using proteomics approaches is promising since it can be used to identify body fluids and tissues, as well as to convey genetic information in the form of SAAVs as the result of nsSNPs (Parker et al., 2021). These applications also show a clear example of the potential for identifying individuals considering many of the points introduced in the previous section.

There have been several studies devoted to proteomics genotyping. In this context, the human hair shaft proteome provides a broad representation of the genome to test proteome-based nsSNP imputation for the identification of individuals. In a first pilot forensic study, an analysis of SAAVs of hair shaft proteins was performed using a custom-made protein database [Figure 2, text-box 2, Parker et al., 2016], which contained all SAAVs with a greater than 0.4% allelic frequency in either European-American or African-American sample populations (http://evs.gs.washington.edu/EVS). Overall, more than 35 × 10^3^ nsSNPs with frequencies over 0.8% of the population were considered. The probability of a particular profile occurring in a population was estimated by applying a statistical treatment of the individually imputed nsSNP profiles. The resulting profile of imputed nsSNP alleles enabled a probability estimation of individual non-synonymous SAAV allelic profiles in the European population, with a maximum power of discrimination that a given profile existed of 1 in 12,500 individuals. This allowed performing likelihood measures of biogeographic background. When estimated using a European sample population, the resulting overall profile probabilities ranged from 9.98 × 10^−1^ to 7.21 × 10^−5^.

Subsequent studies of bulk samples were performed on the same number of sample replicates using hair from the same individuals [Figure 2, text-box 4, Mason et al., 2019]. Both single hair samples derived from the same subjects and the bulk-hair samples resulted in an overall higher number of total SAAV identifications. Interestingly, the difference in the standard deviation observed between bulk-hair and single hair samples indicated that in practice, single hair samples may provide different sets of SAAVs depending on body location, hair length or age.

In one more study, it was demonstrated that hair from different body locations could lead to the identification of the same SAAV markers [Figure 2, text-box 3, Chu et al., 2019]. Protein abundance profiles of head and arm hair samples were more similar among themselves than when compared to pubic hair. Additionally, changes in protein abundance were found in 37 markers. This enabled the distinction of hair fibres from different body locations *via* principal component analysis. A different analysis approach was used (Figure 2, text-box 6, Zhang et al., 2020) for the identification of SAAVs in human hair keratin. This involved the construction of a spectral library from hair samples. Overall, the library contained 6,280 spectra, including SAAVs that could be extended with wide-ranging hair-derived peptides. This peptide spectral library contained all identified peptides from their work, including SAAVs that, when expanded with diverse hair-derived peptides, could provide reliable and sensitive means of analysing hair digests. Also, the study showed that genetically variant peptides derived from human hair shaft proteins could be used to differentiate individuals of different biogeographic origins.

In yet another forensic study, human finger-marks were utilised for proteomic genotyping, detecting SAAVs deduced from the matching nsSNPs (Figure 2, text-box 5, Borja et al., 2019). From a total of 264 SNP allele inferences (including 260 true and 4 false positives), 60 SAAVs were validated after matching proteomics and exome sequences, with a PSM FDR of 1.5%. Using data from the Thousand Genomes Project, genotype frequencies from the major matching populations were used to estimate the probability of random matching, which resulted in a value of 1 in 1.7 × 10^8^, with a median probability of 1 in 2.4 × 10^6^. Also, this peptide SAAV detection method enabled the inference of the matching SNP alleles in the donor, as well as in most populations, as proven in the hair studies, and it’s claimed to complement other methods of human identification.

A recent study performed on hair shafts resulted in a deep proteome coverage. The methodology consisted of a three-step ionic liquid-based extraction and two-dimensional reverse-phase LC-MS stages performed at high and low pH. Analysis of SAAV data provided significantly higher numbers in identifications of both variant and reference SAAVs with a maximum power of discrimination of 1 in 3.10 × 10^14^, which appears to be close to the requirements for forensic applications (Figure 2, text-box 9, Wu et al., 2022). In this case, an OMS approach using the software pFind was used. As highlighted above, OMS approaches are not considered, at least currently, as reliable as the typical PG analysis, so in our view, the results of this study should be taken with more caution.

Forensic studies aside, to the best of our knowledge there is only one omics study on this topic in the literature. There, it was shown that MS data collected from the same person could potentially be used to reidentify individuals using nsSNPs (Figure 2, text-box 1, Li et al., 2016). Overall, 158 proteomics samples were collected coming from the serum of 80 breast cancer patients and 78 healthy individuals (used as controls), as well as an additional set of blood/serum samples coming from 30 individuals. The detected nsSNP sites showed up to 20 minor allelic variant sites corresponding to 25 mass spectra. The minor allele frequencies indicated that the participants could be correctly reidentified with high confidence (*p*-value<10^–10^).

### Identifiability risks of other proteomics data types

In addition to protein sequence variation, the risk of identifying individuals is also present in other proteomics data types. Peptide and protein expression/abundance values (analogously to gene expression values) have already been studied in this context. As clinical proteomics can generate analyses of large-scale cohorts of e.g., plasma protein levels, it can usually report on many more biological conditions than the main one under examination, making ‘Incidental Findings’ an integral feature of the approach. A recent study describing a meta-analysis of clinical plasma proteome datasets revealed that individual-specific protein expression values could be used to reidentify the individuals, and additionally, also found some incidental findings that had ethical considerations (Figure 2, text-box 7, Geyer et al., 2021). Individual protein expression levels depend on several factors such as age and lifestyle, however, proteomics data can provide broad insight into linking observed proteins to genetic and phenotypic features such as ethnicity, gender, and disease. Furthermore, proteomics findings have revealed that the abundance of proteins involved in the same biological process varies among individuals. It appears that these processes are tightly regulated at the protein level (Wu et al., 2013). Additionally, some studies have observed a significant difference between the proteomes of different genders and their reproductive states. As an example, females have an increased prevalence of oestrogen-regulated proteins, such as sex hormone binding globulin (SHBG) and pregnancy zone protein (PZP), which are detectable using MS-based proteomics. SHBG and especially PZP further increase more than tenfold during pregnancy (Moore & Dveksler, 2014; Gordon et al., 1977). MS-based detection of Vitamin-D binding protein [DBP, also known as Group-specific Component (GC)] allelic types and abundance in plasma abundance has been linked to ethnicity. The gene encoding this protein has three common alleles Gc1f, Gc1s, and Gc2, each with very different allele distributions and protein expression profiles depending on ethnic background. Gc1f is most frequently found in West Africans and African Americans, and least common in Caucasians (Kamboh and Ferrell, 1986; Constans et al., 1985).

Proteomics, like genomics data, can predict disease risks which can influence personal decisions (i.e., concerning insurance, jobs, family planning, or other lifestyle choices) in helping minimise disease development. However, proteomics-based diagnoses involving medically unactionable information could have a significant negative impact on patients, leading to unnecessary medical procedures and mental health considerations. One example is the case of the three APOE alleles (APOE2, APOE3, and APOE4), which can be differentiated by sequence-specific peptides. Detection of the APOE4 allelic peptide (present in 7.5%–15.6% of the population) represents a non-actionable biomarker-related condition, strongly indicating a significantly increased risk of Alzheimer’s disease (Mckay et al., 2011). However, actionable knowledge about the APOE2 allele (6.7%–10.0% of the population), linked to increased cholesterol levels and cardiovascular pathologies (Mckay et al., 2011), allows early intervention and medical treatment. Another example is the glycated form of Haemoglobin HbA1c, an actionable biomarker of diabetes. It is easily detectable by plasma proteome profiling experiments, and highly relevant for a third of the population. Finally in this context, the U.S. Food and Drug Administration (FDA) has approved 50 proteins as health status biomarkers, which can be identified by MS-based proteomics (Geyer et al., 2016). These MS-quantified markers include the C-reactive protein (CRP) and the protein serum amyloid alpha 1 (SAA1) with abundance changes linked to infection and an individual’s inflammation status. Both are protein measurements that are highly requested in clinical practice.

The same principles used for peptide and protein expression abundance could also be applied to peptides/proteins with differential PTM profiles that can be associated with disease phenotypes in individuals. This topic has recently been extensively reviewed by others in cancer (Zhu et al., 2022), or in neurodegenerative diseases (Azevedo et al., 2022) such as Alzheimer’s disease (Karikari et al., 2022). It is also worth highlighting a study where differential PTM-based proteoform profiles were detected using top-down proteomics, which was derived from underlying SAAVs characteristic of different individuals (Lin et al., 2019).

### Future perspectives

Analysing (clinically sensitive) human proteomics data might lead to the identification of individuals, raising ethical concerns. At present, in our view, the identification of an individual remains very challenging and unlikely. However, matching two datasets or matching a proteomics dataset to a DNA/RNA sequencing one, based on a set of identified SAAVs, can be feasible. Despite this, several methodologies are continuously improving SAAV detection, and new algorithms are being developed, involving for instance analysis approaches without using sequence databases or experimentally generated spectral libraries. By detecting SAAVs, rather than using only genomic sequencing data, it is possible to better predict disease risk considering Proteomics SAAV data. For instance, SNPs associated with disease risk may affect the expression of disease protein biomarker(s) when these SNPs are properly identified or quantified as SAAVs. This medically actionable or unactionable information is critical as it may result in potentially negatively influencing future personal decisions.

In addition to other parameters, phenotypes derived from proteomics data must be evaluated regarding their risk for identifiability, considering which minimal information would be required to assess the identifiability potential. Likewise, data standards and archiving practices in the field will need to evolve to comply with the state-of-the-art in other omics disciplines such as genomics and transcriptomics, including for instance the availability of controlled-access proteomics data repositories (Keane et al., 2021). Indeed, the datasets available in current proteomics repositories (which are completely open), could be reanalysed as new methods arise, creating future risks in terms of identifiability even if these risks were not apparent at the time of the data submission.

To finalise, it should be reiterated that inherent ethical and privacy issues must be formally considered in the proteomics field. In that context, in our view, it is critical that larger-scale studies can be conducted to gain a deeper understanding of the identifiability risks associated with the different proteomics data types and approaches.

## Abbreviations

DDA, Data Dependent Acquisition; DIA, Data Independent Acquisition; FDR, False Discovery Rate; MS, Mass Spectrometry; OMS, Open Modification Search; PG, Proteogenomics; PSM, Peptide Spectrum Match; SAAV, Single Amino Acid Variant; SNP, Single Nucleotide Polymorphism; LC-MS, Liquid chromatography–mass spectrometry.
